# Experimental Guesswork with Quantum Side Information Using Twisted Light

**DOI:** 10.3390/s23146570

**Published:** 2023-07-21

**Authors:** Vishal Katariya, Narayan Bhusal, Chenglong You

**Affiliations:** 1 Hearne Institute for Theoretical Physics, Department of Physics & Astronomy, and Center for Computation & Technology, Louisiana State University, Baton Rouge, LA 70803, USA; 2 Quantum Photonics Laboratory, Department of Physics & Astronomy, Louisiana State University, Baton Rouge, LA 70803, USA

**Keywords:** quantum information, quantum optics, quantum cryptography

## Abstract

Guesswork is an information–theoretic quantity which can be seen as an alternate security criterion to entropy. Recent work has established the theoretical framework for guesswork in the presence of quantum side information, which we extend both theoretically and experimentally. We consider guesswork when the side information consists of the BB84 states and their higher-dimensional generalizations. With this side information, we compute the guesswork for two different scenarios for each dimension. We then performed a proof-of-principle experiment using Laguerre–Gauss modes to experimentally compute the guesswork for higher-dimensional generalizations of the BB84 states. We find that our experimental results agree closely with our theoretical predictions. This work shows that guesswork can be a viable security criterion in cryptographic tasks and is experimentally accessible in a number of optical setups.

## 1. Introduction

Guesswork is an information–theoretic measure of security and uncertainty of an information source [1,2], similar to entropy. In its simplest form, it can be understood as a game between two agents, Alice and Bob. Alice picks an element *x* from an alphabet X with prior probability $p_{X}\left(x\right)$. Bob’s task then is to guess Alice’s choice *x* while being allowed to ask questions in the form of “Is X=x?”. The guesswork, G(X), is the average number of guesses Bob needs until Alice answers with “yes”. This is in contrast to entropy, where the same game is played, except that Bob is allowed to ask questions in the form of “Is $X\in \tilde{X}?$”, where $\tilde{X}$ is a subset of the alphabet X [3].

Guesswork also has real-world applicability. Consider that one’s account on an online portal is subjected to a brute-force hacking attack. A malicious agent is only allowed a certain number of guesses of the password before being locked out. The average number of guesses, i.e., the guesswork, would be the operational criterion of security in such a situation. Furthermore, guesswork takes on richer behavior when Bob possesses some quantum correlations with Alice, also known as side information. The theoretical framework for this problem has been laid out and studied recently [4,5,6,7,8,9]. For a general quantum ensemble, guesswork was shown to be computable by a semidefinite program [4,5]. Closed-form expressions for certain special cases of the guesswork exist, in particular, ensembles of qubit states [6,7,8], and the extension of guesswork to the study of classical-quantum channels has been recently initiated in Ref. [9]. However, experimental verification of the guesswork with quantum side information is yet an unexplored avenue.

Recently, spatially structured beams of light have been used extensively for multiple applications, such as 3D surface imaging, quantum cryptography, remote sensing, and correlated imaging [10,11,12,13,14,15,16,17,18,19,20,21]. Among them, the Laguerre–Gauss (LG) modes are particularly important, as they possess orbital angular momentum (OAM) [22,23] and allow the construction of OAM modes of light. OAM modes enable the construction of orthonormal bases of light in any arbitrary finite dimension. OAM also enables the construction of a mutually unbiased basis of azimuthal angle (ANG) [24,25,26]. These two properties allow for the generation of the qubit BB84 states [27], as well as their higher-dimensional generalizations, which are especially important in quantum cryptography.

In this work, we perform a proof-of-principle experiment in which we use spatial modes of light to experimentally calculate and verify the value of guesswork for several physically and cryptographically relevant examples involving the BB84 states. We extend the work of Refs. [5,6], both theoretically and experimentally, to higher-dimensional generalizations. Generalizing to higher dimensions allows for an enlarged alphabet and, thus, a more versatile and secure protocol, which is especially attractive considering that the same beams of light are used. We find excellent agreement between our experimental results and theoretical predictions. In each of the cases considered, we find that there is a “quantum” gap in the guesswork between the standard basis measurement and the optimal projective measurement. Our work shows that guesswork can be a viable security criterion in cryptographic tasks, and is experimentally accessible from optical setups.

## 2. Theory

**Figure 1:**
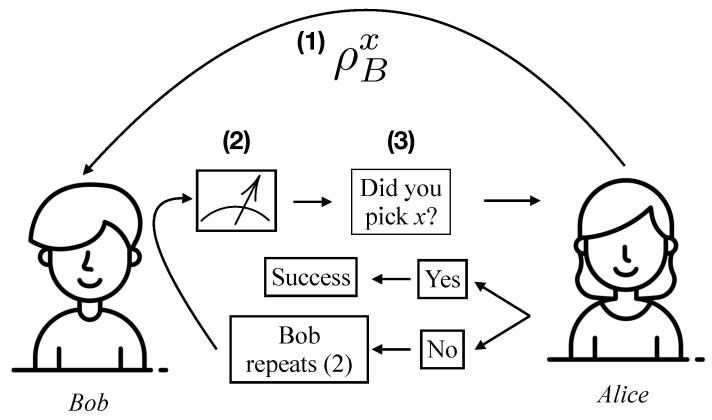
Guesswork can be understood via this guessing game played by Alice and Bob. Alice picks a classical symbol *x* and sends Bob the corresponding quantum state. Bob guesses Alice’s symbol with the help of his quantum state $\rho_{B}^{x}$. In each round of the game, Bob performs a quantum measurement and uses the classical outcome to make a guess. He repeats this process until he guesses correctly.

First, we introduce the theoretical framework of guesswork with quantum side information. Guesswork with quantum side information can be viewed as a multi-round two-party game, as shown in Figure 1. The guesser, Bob, has a classical system or, more generally, a quantum system *B*, which is correlated with Alice’s random variable *X*. In this work, we consider the latter (and more general) case where Bob possesses quantum side information. This scenario is fully characterized by a classical quantum state $\rho_{XB}$ shared by Alice and Bob, where Alice’s symbols and their associated probabilities are captured in the classical register *X*, and Bob’s quantum side information is present in the quantum system *B*. In the guessing game picture of guesswork, Alice picks an element x∈X and sends Bob the corresponding quantum state $\rho_{B}^{x}$. Bob then performs quantum measurements on his side information state $\rho_{B}^{x}$, the results of which inform his guessing strategy. In general, Bob performs a quantum instrument before each guess. A quantum instrument yields both a classical measurement outcome and a post-measurement quantum state. This ensures that after each round, Bob has classical information used to make a guess, and also a quantum state for future rounds of the guessing game.

However, it was shown in Ref. [5] that Bob can do just as well if he performs a single quantum measurement to decide his guessing order; that is, the typical sequential guessing strategy can be reduced to a single-round guessing strategy. This divides the guessing game into two parts: an initial step involving a quantum measurement, followed by a purely classical guessing game between Alice and Bob. Such an equivalence makes a trade-off between time and space, in the sense that Bob needs more spatial resources and fewer temporal resources than the sequential guessing game.

A simple and yet instructive example of guesswork with quantum side information is that involving the four BB84 states [27]. In this case, Alice first picks one of four classical letters $x_{1}$ through $x_{4}$ with equal probability, and then sends the corresponding BB84 states {|0〉,|1〉,|+〉,|−〉} to Bob. Therefore, Bob’s side information is hidden in his quantum state. Bob’s task is to use his received quantum state to guess which classical letter Alice chose. Suppose that the projective measurement is characterized by the two orthogonal states {|ψ(θ)〉,|ψ(π/2−θ)〉} where |ψ(θ)〉:=cosθ|0〉+sinθ|1〉. Alice’s states constitute mutually unbiased bases, and Bob’s naive strategy would to be to measure in one of them. This would correspond to measuring in the {|0〉,|1〉} basis, or the standard basis as it is known. Measuring in this basis offers the scenario most similar to a classical or digital measurement and yields the average number of guesses as 1.75 (See Appendix A). However, there exists an optimized projective measurement which leads to a smaller guesswork. This optimal measurement is characterized by θ=1/2arctan(1/3). This measurement can be shown to achieve a guesswork of 1.709 [5,6].

It was shown in Ref. [6] that for this case, and in general for any qubit ensemble with uniform probability distribution, a projective measurement suffices to attain the guesswork. Corollary 2 of Ref. [6] provides a closed-form expression for the guesswork applicable in this case, which evaluates the guesswork to be $\frac{5}{2}-\sqrt{\frac{5}{8}}=1.709$, which, for the above-discussed BB84 example, is indeed the value obtained by the optimized projective measurement characterized above by θ=1/2arctan(1/3). Projective measurements achieve the minimum guesswork only in the d=2 case, and are not sufficient in higher dimensions [6].

From the above simple example, we see a clear separation of guesswork when using the optimal projective measurement compared to a “standard” basis measurement. Such a separation can be interpreted as a quantum gap, or quantum advantage, as an optimized quantum measurement results in lesser number of guesses as compared to a standard basis measurement (resembling a classical/digital measurement). Such a “quantum” separation of the guesswork can also be obtained in higher-dimensional generalizations of the BB84 example; that is, we consider the side information system in the *d*-dimension BB84 generalization. Alice will pick one of the 2d classical symbols with equal probability, each of which is correlated with one of the 2d side information states. These 2d states are divided into two mutually unbiased bases of *d* states each. The states are as follows:

(1) $$\left\{|0\rangle ,|1\rangle ,\ldots ,|d-1\rangle ,|\tilde{0}\rangle ,|\tilde{1}\rangle ,\ldots ,|\tilde{d-1}\rangle \right\}$$

where $|\tilde{j}\rangle =\frac{1}{\sqrt{d}}\sum_{k=0}^{d-1}e^{i2\pi kj/d}|k\rangle$ and ${\left|\langle i|\tilde{j}\rangle \right|}^{2}=1/d\;\forall \;i,j\in \left\{0,1,\ldots ,d-1\right\}$. In this case, a standard basis measurement by Bob means that he projects his state onto the basis {|0〉,|1〉,…,|d−1〉}. When outcome |k〉 is obtained, Bob can eliminate the d−1 standard basis states that are orthogonal to |k〉. His best strategy, in this case, is then to guess outcome *k* first, then $\tilde{0}$ through $\tilde{d-1}$, and finally the remaining labels in any order. The guesswork in this case is (d+5)/4. We provide more details of this calculation in Appendix A.

However, like in the two-dimensional case, Bob can do better by carefully selecting his quantum measurement. We briefly explain the strategy he can use and how it can be optimized. Consider that Bob chooses to project onto an arbitrarily chosen orthonormal basis $\left\{|\psi_{0}\rangle ,\ldots ,|\psi_{d-1}\rangle \right\}$. If he obtains the outcome corresponding to $|\psi_{k}\rangle$, then he guesses in decreasing order of the overlap between $|\psi_{k}\rangle$ and the 2d input states. We note again here that the post-measurement guessing strategy is purely classical, and we obtain it by invoking Massey’s observation [1] to minimize the guesswork by guessing in decreasing order of the posterior probability of classical symbols.

Since the rules of the game are decided beforehand, Bob finds and decides on his optimal projective measurement via a numerical technique. We perform this optimization for dimensions d=3 and 4 using MATLAB. This optimization also yields the guessing order to use with each of these measurements. Using this technique, we find that there is a significant gap between the guesswork attained by standard basis measurements and that attained by the optimized projective measurement. We summarize this gap in Table 1 and provide more details in Appendix B. We note again that the optimized projective measurement does not yield the minimum guesswork, as we are not optimizing over all POVMs, yet does provide a lower guesswork than the standard basis measurement. Simulating these projective measurements with twisted light can be done by using holograms generated by a spatial light modulator (SLM), which is what we use in our experiment, described below.

## 3. Experiment

We now proceed to describe the experimental apparatus and techniques used to perform our experiment. The generalized BB84 states consist of two mutually unbiased bases. The bases we use in our experiment are the OAM basis and the ANG basis, which are mutually unbiased. Each mode of light in the OAM basis is characterized by an angular momentum quantum number *ℓ*. In principle, *ℓ* can be any integer and, therefore, the OAM basis consists of an infinite number of orthogonal modes; thus, it is simple to generate an orthonormal basis in arbitrary dimensions by selecting the appropriate values of *ℓ*.

For example, if Alice generates the OAM basis consisting of qunatum numbers ℓ∈{−L,−L+1,…,L−1,L}, then we have a d=2L+1-dimensional OAM basis, consisting of the states ${\left\{\Psi_{\mathrm{OAM}}^{\ell }=e^{i\ell \varphi }\right\}}_{\ell =-L}^{\ell =L}$.

The ANG basis corresponding to the OAM basis defined above also consists of 2L+1 orthogonal states. Each ANG state is a superposition of each of the OAM states. The basis is constituted by the following states:

(2) $${\left\{\Psi_{\mathrm{ANG}}^{n}=\frac{1}{\sqrt{d}}\sum_{\ell =-L}^{\ell =L}e^{\frac{2i\pi n\ell }{d}}\Psi_{\mathrm{OAM}}^{\ell }\right\}}_{n=-L}^{n=L}.$$

Simple verification shows that the two bases are indeed mutually unbiased, i.e.,

(3) $$|\langle \Psi_{\mathrm{OAM}}^{l}|\Psi_{\mathrm{ANG}}^{n}\rangle {|}^{2}=1/d.$$

### 3.1. Experimental Setup

**Figure 2:**
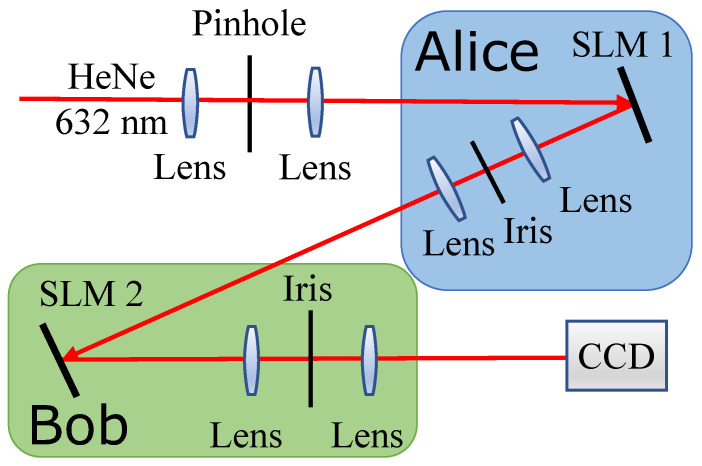
The schematic diagram of the setup used to demonstrate guesswork with quantum side information. The experiment utilizes a He-Ne laser whose output spatial mode is first cleaned. The higher-dimensional generalizations of the BB84 states are prepared by Alice using a spatial light modulator (SLM). The prepared states are sent to Bob through a free-space communication channel, a 4f system. Bob then performs his quantum measurement using a second SLM and a charged coupled device (CCD) camera.

The schematic diagram of our experimental setup is depicted in Figure 2. Here, Alice prepares quantum states of light which corresponds to her choice of symbol, and then sends to Bob for further processing. In our experiment, Alice uses a spatial light modulator (SLM) and computer-generated holograms to generate the LG modes required in our experiment [28]. This technique enables us to generate spatial modes in the first-order diffraction order of the SLM. This is sufficient to generate the OAM and ANG modes that correspond to generalized BB84 states in higher dimensions. It is also a characteristic of using such spatial modes of light that the same results will hold when the input beams of light consist of single photons, similar to the results of Ref. [29]. The generated modes are filtered and sent to Bob using a 4f optical system. Bob then projects the spatial mode onto a second SLM to perform a projective measurement. The specific holograms imprinted on the SLM dictate the basis onto which the initial mode is projected. The beam reflected by the second SLM corresponds to a post-measurement state, which is propagated to a charge-coupled device (CCD). The spatial profile of the final beam is captured and analyzed. This process is repeated for each of the measurement states to be projected on, and the captured images are then used to compute the guesswork.

### 3.2. Experimental Determination of Guesswork

Our goal is to compute the guesswork for the generalized BB84 states for dimensions d=2,3, and 4. For each dimension, the guesswork is computed for the standard basis measurement, as well as the optimized projective measurement, which makes for a total of six different scenarios. We remark here again that measuring in the standard basis, in this case, one of the mutually unbiased bases comprising the input states, is most akin to a classical measurement and the optimized projective measurement would represent a quantum advantage.

In each iteration of the guessing game, Bob begins with a predecided guessing order for each possible measurement outcome. For each state Alice sends, he projects onto each of the basis states that characterize his measurement. This enables him to determine the relative rate of the measurement outcomes and, hence, decide on the measurement outcome. This holds for both the standard and optimized basis measurement. Once the guessing order is decided, Bob simply sends Alice his guesses—this interaction yields the average number of guesses for each input state. Averaging over input states yields the overall guesswork. The post-processing of CCD images to compute the guesswork is performed using MATLAB.

We perform a total of six experiments corresponding to six scenarios: this comprises two sets of measurements for each of the three dimensions considered. The two measurements are the standard basis measurement and the numerically determined optimal projective measurement. In each case, we repeated the measurement ten times for each input state. Overall, this is equivalent to playing the guesswork game ten times for each of the six scenarios under consideration.

In Table 1, we present our experimental results. The results are divided into two parts: the guesswork when performing a standard basis measurement, and when performing the optimized projective measurement. The errors reported correspond to the standard deviation of our data across experimental iterations. We see that for all scenarios we consider, the guesswork is within 1% of the theoretical prediction for both the standard basis and optimal measurement.

We note here that for such a protocol to be deployed in a real-world setting, there are other considerations that are to be taken into account. These include, but are not limited to, the vulnerability of the protocol to side-channel attacks, such as fault attacks, power analysis attacks, and even combined differential fault analysis and differential power analysis. Specific countermeasures, such as fault detection architectures [30], error detection schemes [31], or fault diagnosis schemes [32], will need to be built in to a security protocol to guard against these attacks. Such a protocol could be implemented either on a field-programmable gate array (FPGA), an application-specific integrated circuit (ASIC), or on an ARM processor, to name a few. The side channel attack evaluation for each of these will be slightly different, e.g., the difference between the results of Refs. [30,33]. The advent of post-quantum cryptography schemes will also need to be considered, with implementations such as those in Refs. [33,34].

**Figure 3:**
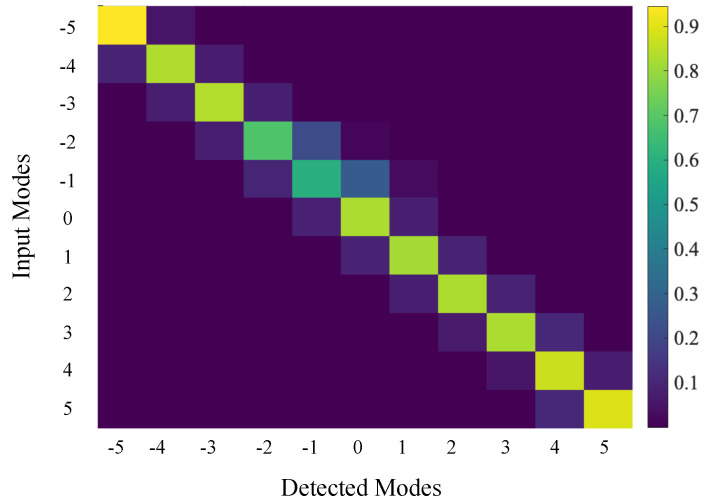
The cross-correlation matrix representing the conditional probabilities between sent and detected modes in the OAM basis. The off-diagonal elements in the figure indicate cross-talks between adjacent modes. The experimental overlap of two adjacent OAM modes is small, indicating a good selection of OAM modes for the experiment.

Finally, we remark on the choice of specific OAM modes used for each dimension *d*. OAM modes are, in principle, orthogonal to each other; thus, any combination of them can be used to construct the desired orthonormal basis. However, due to the finite size of the SLM pixels, they are not perfectly orthogonal in practice. We quantify the overlap between the OAM modes under consideration using a cross-correlation matrix, shown in Figure 3. To minimize overlap errors in our experiment, we choose *ℓ* values that are spaced further apart from one another to generate our input states, instead of using consecutive *ℓ* values. For d=2, we use the *ℓ* values {−3,3}. Similarly, for d=3 and d=4, we use *ℓ* values {−3,0,3} and {−3,−1,1,3}, respectively. We note that this modification does not alter any of the calculations for the guesswork itself, and is done only to reduce errors that arise from non-zero overlap between nearby OAM modes. An illustrative demonstration of the input modes used for d=3, as well as some of the post-measurement states, are provided in Appendix C.

## 4. Conclusions

In summary, we showed how to use accessible spatial modes of light to experimentally compute the guesswork in the presence of side information. We considered the side information to be higher-dimensional generalizations of the qubit BB84 states, and showed that the experimentally calculated guesswork matches the theoretical predictions to within an error of 1%.

This proof-of-principle work lays the ground for further experimental applications of guesswork with quantum side information. Ref. [9] identifies a number of use-cases for using the guesswork as an operational quantifier of information, such as in information–disturbance relations and majorization theory. Experimental verification will be necessary for theoretical results that address these problems. The avenues of research for future experimental work also include identifying and performing the optimal measurement for each scenario outside of projective measurements, performing vulnerability analysis for various side channel attacks, and devising countermeasures to such attacks. We hope that our work will serve as a starting point for more experimental uses of the guesswork as a security criterion.

## Figures and Tables

**Table 1:** The guesswork for each of the scenarios considered.

Dimension	Theoretical Value	Experimental Value
Standard basis measurement		
*d* = 2	1.75	1.7505±0.0017
*d* = 3	2	1.9996±0.0087
*d* = 4	2.25	2.2547±0.0029
Optimized projective measurement		
*d* = 2	1.709	1.7062±0.0089
*d* = 3	1.9425	1.9439±0.0084
*d* = 4	2.1429	2.1411±0.0025

**Figure A1:**
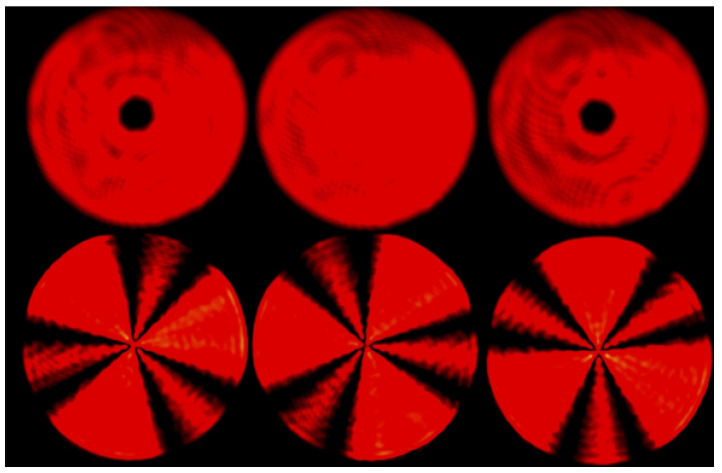
The alphabet of input states for the particular case of dimension d=3. Each image is the CCD image of one of the six input states used. The first row corresponds to OAM modes with l=−3, l=0, and l=3. The second row corresponds to the corresponding ANG modes which are created by performing uniform superpositions of the OAM modes in the first row.

**Figure A2:**
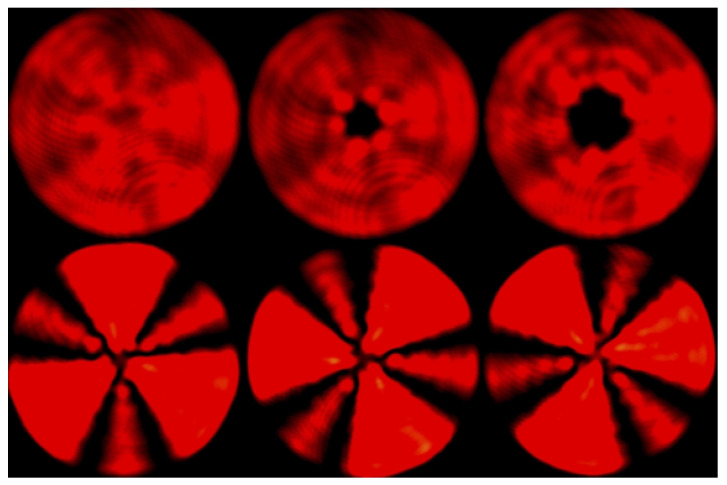
The figure shows the six states in Figure A1 when projected onto the first state of the optimized projective measurement basis, i.e., the state characterized by Equation (A11).

## Data Availability

Data supporting the reported results is available on request.

## Appendix A. Guesswork Calculation with a Standard Basis Measurement

### Appendix A.1. d = 2

In the main text, we stated that when the side information consists of the four BB84 states, the average number of guesses is 1.75 when Bob performs a standard basis ({|0〉,|1〉}) measurement.

This number can be understood as follows: if the measurement outcome is |0〉, then the posterior probabilities of states {|0〉,|1〉,|+〉,|−〉} are {1/2,0,1/4,1/4}. Likewise, for outcome |1〉, the posterior probabilities are {0,1/2,1/4,1/4}. For outcome |0〉, Bob guesses in the order $\left(x_{1},x_{3},x_{4},x_{2}\right)$ and for outcome |1〉, the guessing order is $\left(x_{2},x_{4},x_{3},x_{1}\right)$. In each of these cases, the average number of guesses is 1·1/2+2·1/4+3·1/4 and, thus, the guesswork $G{\left(X|B\right)}_{\rho }=1.75$.

### Appendix A.2. General Dimension d

In the main text, we showed that for the higher-dimensional generalization of the BB84 example, the guesswork when using a standard basis measurement is (d+5)/4 for dimension d≥2. Here, we show how this is obtained.

We recall that, for dimension *d*, the 2d side information states are divided into two mutually unbiased bases of *d* states each:

(A1) $$\{|0\rangle ,|1\rangle ,\ldots ,|d-1\rangle ,|\tilde{0}\rangle ,|\tilde{1}\rangle ,\ldots ,|\tilde{d-1}\rangle \}.$$

When Bob performs a standard basis measurement, it means that he projects his state onto the basis {|0〉,|1〉,…,|d−1〉}. We know from Massey’s criterion [1] that, for optimizing the guesswork, Bob should guess in decreasing order of the posterior distribution of measurement outcomes. When outcome |k〉 is obtained, Bob can eliminate the d−1 computational basis states that are orthogonal to |k〉.

Given measurement outcome corresponding to |k〉, the posterior probabilities of the states

(A2) $$\left\{|0\rangle ,|1\rangle ,\ldots ,|k\rangle ,\ldots ,|d-1\rangle ,|\tilde{0}\rangle ,|\tilde{1}\rangle ,\ldots ,|\tilde{d-1}\rangle \right\}$$

are {0,0,…,1/2,…,0,1/2d,1/2d,…,1/2d}. This means that Bob’s guessing order is $\left(x_{k},x_{\tilde{0}},\ldots ,x_{\tilde{d-1}},x_{0},\ldots x_{k-1},x_{k+1},\ldots x_{d-1}\right)$. We note here that this guessing order is quite flexible—all that is required is that the first guess by $x_{k}$; the next *d* guesses correspond to $x_{\tilde{0}}$ through $x_{\tilde{d-1}}$ in any order, and the remaining standard basis symbols follow again in any order.

The probability of Bob’s first guess being right is 1/2, which we read off the posterior distribution. In case it is incorrect, then the probability of correctness of each of his next *d* guesses is 1/2d. We can use this to compute the expected value of the number of guesses:

(A3)E[G(X|B)]=1×12+2×12d+⋯+(d+1)×12d+0+⋯+0(A4)=12+12d2+3+⋯+(d+1)(A5)=12+12d(d+1)(d+2)2−1(A6)=14d(2d)+14dd2+3d(A7)=d+54.

## Appendix B. Guesswork Calculation with the Optimal Projective Measurement

First, we detail how we optimize over all projective measurements for arbitrary dimension *d*. To do so, we consider the basis $\left\{|\psi_{0}\rangle ,\ldots ,|\psi_{d-1}\rangle \right\}$ to arise from a parameterized d×d unitary matrix [35]. The rows of the matrix correspond to the coefficients of each state of the orthonormal basis. The guessing orders for each measurement outcome are obtained by calculating the overlaps of each basis state with the generalized BB84 states and then arranging the generalized BB84 states in descending order of their overlaps, just like we described for the standard basis measurement in the preceding paragraph. The optimization procedure to determine the optimal measurement is straightforward, and is performed using the Global Optimization Toolbox in MATLAB. As an illustrative example, we describe the details of this calculation for d=3 below.

### Appendix B.1. Guesswork Calculation for d = 3 with the Numerically Determined Measurement

In Table 1 of the main text, we stated that the guesswork for the d=3 BB84 example was 2 when using the standard basis measurement. This follows the (d+5)/4 formula that we derived just above in (A7). We also state that using a numerically predetermined projective measurement, we can do better and attain a value of 1.9425. Here, we show how this is achieved.

We recall that the six side information states are:

(A8) $$\left\{|0\rangle ,|1\rangle ,|2\rangle ,|\tilde{0}\rangle ,|\tilde{1}\rangle ,|\tilde{2}\rangle \right\}$$

whose corresponding classical symbols are $x_{0},x_{1},x_{2},x_{\tilde{0}},x_{\tilde{1}},\;\mathrm{and}\;x_{\tilde{2}}$.

Our numerical goal is to optimize the overall possible projective measurements with the aim of minimizing the guesswork; thus, we consider an arbitrary orthonormal basis $\left\{|\psi_{0}\rangle ,|\psi_{1}\rangle ,|\psi_{2}\rangle \right\}$ onto which we project.

Given an arbitrary basis $\left\{|\psi_{0}\rangle ,|\psi_{1}\rangle ,|\psi_{2}\rangle \right\}$, the first step is to calculate the overlaps of each of the six side information states (A8) with the three states given above. For each of $|\psi_{0}\rangle$, $|\psi_{1}\rangle$, and $|\psi_{2}\rangle$, we arrange the side information states in decreasing order of overlap. This gives us the optimal guessing order corresponding to each measurement outcome, and the overlaps for each measurement outcome yield the posterior distribution of the correct answer; thus, the average number of guesses for each measurement outcome can be calculated.

Further, Bob’s state alone, before any measurement is done, is the maximally mixed state. This can be seen by considering the states in (A8) and taking a uniformly distributed mixture of them. This means that each measurement outcome, averaged over input states, is equally likely. Therefore, the overall guesswork is the average of the number of guesses for each measurement outcome.

So far, we have described how to calculate the guesswork corresponding to an arbitrarily chosen measurement basis $\left\{|\psi_{0}\rangle ,|\psi_{1}\rangle ,|\psi_{2}\rangle \right\}$. All that remains is to optimize over all such measurement bases. These states are parameterized by considering a parameterization of a d×d unitary matrix given by Hedemann [35]. A 3×3 unitary matrix is parameterized by six complex parameters, *a* through *f*, in the following manner:

(A9) $$U=\left(\begin{array}{ccc} a & bc & bd\\ b^{*}e & -a^{*}ce-d^{*}f^{*} & -a^{*}de+c^{*}f^{*}\\ b^{*}f & -a^{*}cf+d^{*}e^{*} & -a^{*}df-c^{*}e^{*} \end{array}\right),$$

subject to the constraints ${\left|a\right|}^{2}+{\left|b\right|}^{2}=1$, ${\left|c\right|}^{2}+{\left|d\right|}^{2}=1$, and ${\left|e\right|}^{2}+{\left|f\right|}^{2}=1$.

We, thus, have that:

(A10) $$\begin{array}{cc} |\psi_{0}\rangle & =a|0\rangle +bc|1\rangle +bd|2\rangle \\ |\psi_{1}\rangle & =b^{*}e|0\rangle +\left(-a^{*}ce-d^{*}f^{*}\right)|1\rangle +\left(-a^{*}de+c^{*}f^{*}\right)|2\rangle \\ |\psi_{2}\rangle & =b^{*}f|0\rangle +\left(-a^{*}cf+d^{*}e^{*}\right)|1\rangle +\left(-a^{*}df-c^{*}e^{*}\right)|2\rangle . \end{array}$$

We use MATLAB’s Global Optimization Toolbox to optimize over the six variables and three constraints provided above, and calculate the optimal guesswork for this example across all projective measurements. The optimal states are given by the following coefficients:

(A11) $$\begin{array}{cc} |\psi_{0}\rangle & =\left(0.7413-0.6421i,\;0.0118+0.1221i,\;-0.1085-0.1067i\right) \end{array}$$

(A12) $$\begin{array}{cc} |\psi_{1}\rangle & =\left(-0.0919+0.1244i,\;0.0688-0.0060i,\;-0.8069-0.5659i\right) \end{array}$$

(A13) $$\begin{array}{cc} |\psi_{2}\rangle & =\left(0.0676+0.0985i,\;0.9847+0.1023i,\;0.0634+0.0389i\right). \end{array}$$

To calculate the guesswork from this, we construct the overlap table between the side information states and the measurement states as follows:

**Table A1:** This table shows the overlap between each of the input states and each of the states characterizing the measurement. Each row corresponds to an input state and each column corresponds to one of the measurement states.

	$\psi_{0}$	$\psi_{1}$	$\psi_{2}$
|0〉	0.4809	0.0120	0.0071
|1〉	0.0075	0.0024	0.4901
|2〉	0.0116	0.4857	0.0028
$|\tilde{0}\rangle$	0.2574	0.1170	0.1257
$|\tilde{1}\rangle$	0.1347	0.1482	0.2171
$|\tilde{2}\rangle$	0.1079	0.2348	0.1572

For each measurement outcome, the number of guesses can be calculated by arranging the corresponding column in descending order, and then taking an inner product of the column with $\left(1\;2\;3\;4\;5\;6\right)$. We then get that the average number of guesses for each of the measurement outcomes is 1.9344, 1.9423, and 1.951. Taking an average of these three numbers yields:

(A14) G(X|B)=1.9425.

### Appendix B.2. Guesswork Calculation for d = 4 with the Numerically Determined Measurement

A similar procedure as above is followed for the case of d=4. Just like we listed out three different states for d=3 in (A10), we have for four dimensions:

(A15) $$\begin{array}{cc} |\psi_{0}\rangle & =a|0\rangle +b^{*}g|1\rangle +b^{*}h^{*}j|2\rangle +b^{*}h^{*}k|3\rangle \\ |\psi_{1}\rangle & =bc|0\rangle +\left(-a^{*}cg+d^{*}hl\right)|1\rangle +\left(-a^{*}ch^{*}j-d^{*}g^{*}jl+d^{*}k^{*}m^{*}\right)|2\rangle \\ \; & +(-a^{*}ch^{*}k-d^{*}g^{*}kl-d^{*}j^{*}m^{*})|3\rangle \\ |\psi_{2}\rangle & =bde|0\rangle +(-a^{*}deg-c^{*}ehl+f^{*}hm)|1\rangle \\ \; & +(-a^{*}deh^{*}j+c^{*}eg^{*}jl-c^{*}ek^{*}m^{*}-f^{*}g^{*}jm-f^{*}k^{*}l^{*})|2\rangle \\ \; & +(-a^{*}deh^{*}k+c^{*}eg^{*}kl+c^{*}ej^{*}m^{*}-f^{*}g^{*}km+f^{*}j^{*}l^{*})|3\rangle \\ |\psi_{3}\rangle & =bdf|0\rangle +(-a^{*}dfg-c^{*}fhl-e^{*}hm)|1\rangle \\ \; & +(-a^{*}dfh^{*}j+c^{*}fg^{*}jl-c^{*}fk^{*}m^{*}+e^{*}g^{*}jm+e^{*}k^{*}l^{*})|2\rangle \\ \; & +(-a^{*}dfh^{*}k+c^{*}fg^{*}kl+c^{*}fj^{*}m^{*}+e^{*}g^{*}km-e^{*}j^{*}l^{*})|3\rangle . \end{array}$$

We use the same optimization technique used for the d=3 case and obtain the final set of measurement states as follows:

(A16) $$\begin{array}{cc} |\psi_{0}\rangle & =\left(-0.1116-0.04115i,\;-0.0015+0.0131i,\;0.1336-0.0510i,\;0.0069+0.9824i\right) \end{array}$$

(A17) $$\begin{array}{cc} |\psi_{1}\rangle & =\left(-0.6943+0.6951i,\;0.05941+0.1301i,\;-0.081+0.0104i,\;-0.1084-0.0490i\right) \end{array}$$

(A18) $$\begin{array}{cc} |\psi_{2}\rangle & =\left(-0.1347+0.0481i,\;0.0138-0.9824i,\;0.1107+0.0435i,\;0.0018-0.0130i\right) \end{array}$$

(A19) $$\begin{array}{cc} |\psi_{3}\rangle & =\left(-0.0104+0.0080i,\;-0.0484+0.1086i,\;0.6991+0.6902i,\;0.1298-0.0602i\right). \end{array}$$

Following the same procedure for calculating the guesswork as we did for d=3, we get the guesswork in this case to be:

(A20) G(X|B)=2.1429.

## Appendix C. Alphabet of Input State Beams Used

As an illustration, we show the input beams used for one of the higher-dimensional BB84 experiments below in Figure A1. It shows the six input states that are used for the d=3 case, where each row of images corresponds to one of the two mutually unbiased bases that make up the six input states.

Further, in Figure A2, we show the profiles of the beams of Figure A1 when projected onto the first basis state of the optimized projective measurement, i.e., Equation (A11).
